# Supplementary material for: Identification of photoperiod-induced specific miRNAs in the adrenal glands of Sunite sheep (Ovis aries)
Source: Front Vet Sci. 2022 Jul 22;9:888207. doi: 10.3389/fvets.2022.888207 (PMC9354845; doi:10.3389/fvets.2022.888207)
Supplement: Supplementary file 1 [file Data_Sheet_1.ZIP › Supplementary file/miRDeep2 file.zip!/miRDeep2 file/Know_Structure/SP42A1/oar-mir-27a.pdf]

miRBase precursor : oar-mir-27a
Total read count : 185968
oar-miR-27a read count : 185720
remaining reads : 248

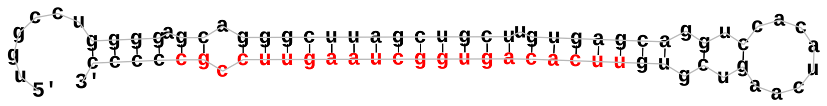

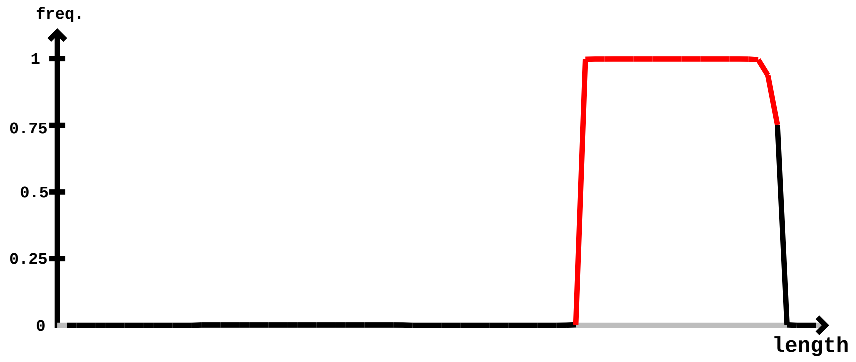

oar-miR-27a

```
5'- uggccuggggagcagggcuuagcugcuugugagcagguccacaucaagucguguucacaguggcuaaguuccgccccc   -3'    exp
    ......((((.((.(((((((((((((.(((((((.(.(........).).))))))))))))))))))).))))))    reads      mm         sample
    .............agggcuuagcugcuugug...............................................    1          0          seq
    .............agggcuuagcugcuugugagc............................................    42         0          seq
    .............agggcuuagcugcuugugagca...........................................    199        0          seq
    .............agggcuuagcugcuugugagcagg.........................................    3          0          seq
    .............agggcuuagcugcuugugagcaggu........................................    2          0          seq
    ..............gggcuuagcugcuugugagca...........................................    1          0          seq
    ...................................................uguucacaguggcuaaguuc.......    4          0          seq
    ...................................................uguucacaguggcuaaguucU......    2          1          seq
    ...................................................uguucacaguggcuaaguucc......    12         0          seq
    ...................................................uguucacaguggcuaaguuccg.....    90         0          seq
    ...................................................uguucacaguggcuaaguuccgc....    8          0          seq
    ...................................................uguucacaguggcuaaguucUgc....    1          1          seq
    ...................................................uguucacaguggcuaaguuccgcc...    3          0          seq
    ....................................................guucacaguggcuaaguucc......    2          0          seq
    ....................................................guucacaguggcuaaguucU......    14         1          seq
    ....................................................guucacaguggcuaaguucUg.....    29         1          seq
    ....................................................guucacaguggcuaaguucUgc....    205        1          seq
    ....................................................guucacaguggcuaaguuccgc....    1          0          seq
    .....................................................uucUcaguggcuaaguu........    1          1          seq
    .....................................................uucacaguggcuaaguG........    1          1          seq
    .....................................................uucacaguggcuaagCu........    2          1          seq
    .....................................................uucacaguggcuaaguu........    45         0          seq
    .....................................................uucacaguAgcuaaguuc.......    1          1          seq
    .....................................................uucacaguggcuaaguuG.......    3          1          seq
    .....................................................uucacagugUcuaaguuc.......    1          1          seq
    .....................................................uucacaguggcuaaguuc.......    424        0          seq
    .....................................................uucacaguggcuaaguucc......    1786       0          seq
    .....................................................uucacaguggcuaaguucU......    9022       1          seq
    .....................................................uucacaguggcuaaguuccA.....    4924       1          seq
    .....................................................uucacaguggcuaaguucUg.....    14168      1          seq
    .....................................................uucacaguggcuaaguuccg.....    15383      0          seq
    .....................................................uucacaguggcuaaguuccgc....    4200       0          seq
    .....................................................uucacaguggcuaaguucUgc....    134905     1          seq
    .....................................................uucacaguggcuaaguuccgcc...    315        0          seq
    .....................................................uucacaguggcuaaguuccgccc..    5          0          seq
```

oar-miR-27a

```
uggccuggggagcagggcuuagcugcuugugagcagguccacaucaagucguguucacaguggcuaaguuccgccccc
......................................................ucacaguggcuaaguuc.......   1      0      seq
......................................................ucacaguggcuaaguucU......   3      1      seq
......................................................ucacaguggcuaaguucc......   2      0      seq
......................................................ucacaguggcuaaguuccA.....   6      1      seq
......................................................ucacaguggcuaaguuccg.....   7      0      seq
......................................................ucacaguggcuaaguucUg.....   7      1      seq
......................................................ucacaguggcuaaguucUgc....   115    1      seq
......................................................ucacaguggcuaaguuccgc....   3      0      seq
.......................................................cacaguggcuaaguucc......   1      0      seq
.......................................................cacaguggcuaaguuccg.....   3      0      seq
.......................................................cacaguggcuaaguuccA.....   1      1      seq
.......................................................cacaguggcuaaguucUgc....   9      1      seq
........................................................acaguggcuaaguuccA.....   1      1      seq
........................................................acaguggcuaaguucUg.....   1      1      seq
........................................................acaguggcuaaguucUgc....   3      1      seq
```
